# Household food insecurity predisposes to undiversified diet in northwest Ethiopia: finding from the baseline survey of nutrition project, 2016

**DOI:** 10.1186/s13104-019-4083-9

**Published:** 2019-01-24

**Authors:** Amare Tariku, Kedir Abdela Gonete, Gashaw Andargie Bikes, Kassahun Alemu, Aysheshim Kassahun Belew, Molla Mesele Wassie, Tadesse Awoke Ayele, Ejigu Gebeye, Zegeye Abebe, Azeb Atnafu Gete, Melkie Edris Yesuf, Yigzaw Kebede, Abebaw Addis Gelagay, Kindie Fentahun Muchie

**Affiliations:** 1 Department of Human Nutrition, Institute of Public Health, College of Medicine and Health Sciences, University of Gondar, Gondar, Ethiopia; 2 Department of Health System Policy, Institute of Public Health, College of Medicine and Health Sciences, University of Gondar, Gondar, Ethiopia; 3 Department of Epidemiology and Biostatistics, Institute of Public Health, College of Medicine and Health Sciences, University of Gondar, Gondar, Ethiopia; 4 Department of Reproductive Health, Institute of Public Health, College of Medicine and Health Sciences, University of Gondar, Gondar, Ethiopia; 5 Department of Epidemiology and Biostatistics, School of Public Health, College of Medicine and Health Sciences, Bahir Dar University, Bahir Dar, Ethiopia

**Keywords:** Adolescent girls, Dietary diversity score, Dabat

## Abstract

**Objective:**

Adolescence represents a critical stage of life, characterized by rapid physical growth and development; varying levels of physical, social and psychological maturity; and a transition from total socio-economic dependence to relative independence. Focusing on adolescents’ nutrition, especially girls, provides a unique opportunity to break the intergenerational cycles of malnutrition. But, there is little information about the dietary diversity of adolescent girls in Dabat district. Therefore, the survey aimed to assess the prevalence and associated factors of dietary diversity among adolescent girls.

**Results:**

The overall prevalence of adequate dietary diversity among adolescent girls was 14.5 (95% CI 12.9, 16.2). The prevalence of adequate dietary diversity among adolescent girls was very low and food insecurity is one of the predisposing factors for low dietary diversity. Therefore, working to enhance household’s food security status is recommended to boost dietary diversification of adolescent’s girls.

## Introduction

Dietary diversity is a qualitative measure of food consumption that reflects household access to a variety of foods and is also a proxy for nutrient adequacy of the diet. The dietary diversity assessment tool is rapid, user-friendly and easily administered low-cost measurement tool [1].

According to the World Health Organization, the adolescent is defined as people whose age ranged between 10 and 19 years [2]. Adolescents constitute 18% (1.2 billion) of the global population [3]. Adolescence marks a critical period of biological and psychosocial growth and development that is unique among phases in the life cycle [4]. Up to 45% of skeletal growth, 15–25% of adult height and 37% of total bone mass are achieved during adolescence [5]. This unique period demands intake of extra nutrients. During the growth spurt of adolescence, up to 37% of total bone mass may be accumulated [6]. Because of this fact, adolescents have increased nutrient demand [7].

In spite of this fact, undiversified diet is commonly noted in poor households [8]. For instance, more than half of adolescent girls and women had low dietary diversity in Bangladesh, and about 70% of participants kilocalories per capita per day from rice alone [9].

In Ethiopia 27% of women were chronic energy deficient (body mass index < 18.5 kg/m^2^), 17% were anemic, and 6% of rural women were experiencing night-blindness in their most recent pregnancy [10].

Malnutrition during adolescence can have lifelong consequences. Gender norms can leave girls disproportionately impacted by food insecurity, but many adolescent boys are malnourished as well and adolescents girls, especially girls in poorer households consume inadequately diverse diets [9]. In developing countries like Ethiopia, there is limited community based studies, assessment, and interventions among adolescent girls.

Despite intensifying efforts to improve adolescent’s dietary diversity and to prevent nutritional problems and its related consequences, the level of diversified diet and its barriers are not well-investigated in Ethiopia. Some available studies were conducted in urban settings which limits the representativeness of the finding [11]. Therefore, our study aimed to assess dietary diversity and associated factors among adolescent girls in Dabat Health and Demography Surveillance System (HDSS) site by addressing the above limitations.

## Main text

### Methods

This study is part of a baseline survey conducted from February to June 2016 for a project entitled ‘Establishing a nutritional surveillance system and piloting nutritional interventions’. This is a 5-year project and has been implemented in Dabat Health and Demographic Surveillance System site, Dabat District, northwest Ethiopia. The site was established in 1996, and currently, it covers a total of 13 kebeles (9 rural and 4 urban kebeles, smallest administration units in Ethiopia) with 17,000 households.

In the baseline survey all pregnant and lactating mothers, children under 5 years and adolescent girls were investigated for varied nutritional, dietary intake, health care utilization, and morbidity characteristics. For this study, all adolescent girls with relevant data were included in the analysis. To check whether the sample size of 1550 enables to estimate adolescent dietary diversity, we used a single population proportion formula to calculate the minimum sample size. Accordingly, the following assumption were considered; a 75.4%, prevalence of adequate dietary diversity, a 95% level of confidence, a 4 margin of error and a 5% non-response rate However, to increase the statistical power we included all eligible adolescents, 1550 adolescent girls.

The structured interviewed administered questionnaire was used for the baseline survey. For this study, a checklist was prepared to select appropriate variables. The outcome variable i.e. dietary diversity was measured using standardized woman dietary diversity score tool. A 24 h recall method was employed. The tool consisted of nine food groups, namely starchy staples, dark green leafy vegetables, other vitamin A rich fruits and vegetables, other fruits and vegetables, organ meat, meat and fish, eggs, legumes, nuts and seeds, and milk and milk products [12]. Considering four food group as a minimum dietary diversity, the total dietary diversity score was classified as adequate if an adolescent girl had four and above dietary diversity score, otherwise, they were deemed having inadequate dietary diversity if their dietary diversity score was less than or equal to three.

Similarly, the household food security status was assessed using standardized tool adopted from Food and Nutrition Technical Assistance (FANTA) 2007.

The baseline survey questionnaire was initially prepared in English and translated into Amharic and retranslated to English with language and public health experts to check the consistency. The questionnaire was pretested in one kebele out of the kebeles under the HDSS site. Experienced 38 data collectors and seven field supervisors who have been permanently working HDSS site were involved in the data collection process. Three days training on interviewing technique and data collection process was given to data collectors and supervisors. Supervisors checked the overall baseline survey on daily basis.

All filled questionnaire were checked manually for completeness and consistency. Epi-data and SPSS version 20 were used for data entry and analysis, respectively. Double data entry carried out to check error during data entry. Descriptive statistics, including frequencies and proportions, were computed and presented using texts, and tables. Both bi-variable and multivariable binary logistic regression analysis were performed. In the bi-variable analysis, variables with a p-value of < 0.2 were considered into the final model and adjusted odds ratio (AOR) with 95% Confidence Interval (CI) was used to show the presence and strength of association. Finally, a p-value of less than 0.05 in the multivariable logistic regression model was used to identify variables significantly associated with dietary diversity.

### Results

#### Socio-demographic characteristics

A total of 1550 adolescent girls were included for analysis, of which 63.6% were in the age range of 10–14 years (early adolescence). A substantial proportion, (88.1%), of adolescents have been attending elementary school while few (4.5%) were not able to read and write. The majority, 86.5 of the adolescents were rural residents. The majority of the adolescents, 89.2%, were being a student in occupation (Table 1).

**Table 1:** Socio-demographic characteristics of adolescent girls in Dabat HDSS site, northwest Ethiopia, 2016

Variables	Response	Frequency	Percentage
Ethnicity	Amhara	1547	99.8
	Others	3	0.2
Religion	Muslim	45	2.9
	Orthodox	1502	96.9
	Others	3	0.2
Ages of the adolescent	Early adolescent (10 to 14)	986	63.6
	Middle adolescent (15 to 17)	502	32.4
	Late adolescent (18 to 19)	62	4
Educational level	Unable to read and write	70	4.5
	Able to read and write	38	2.5
	Elementary	1366	88.1
	High school	74	4.8
	Certificate and above	2	0.1
Marital status of adolescent	Single	1496	96.5
	Married	47	3
	Divorced	7	0.5
Residency	Rural	1340	86.5
	Urban	210	13.5
Occupation	Un employed	20	1.3
	Farmer	37	2.4
	Student	1383	89.2
	Own business	110	7.1
Usual house work			
Fetching water	No	53	3.4
	Yes	1497	96.6
Wood collection	No	357	23
	Yes	1193	77
Farming	No	971	62.6
	Yes	579	37.4
Market	No	869	56.1
	Yes	681	43.9
Mashing	No	748	48.3
	Yes	802	51.7
Baking	No	579	37.4
	Yes	971	62.6
Crushing	No	1120	72.3
	Yes	430	27.7

#### Dietary diversity and other nutrition and health related characteristics

The overall prevalence of adequate dietary diversity among adolescent girls was 14.5% (95% CI 12.9, 16.2). Based on the 24 h dietary recall, almost all. 99.9%, of adolescents ate starchy staples. Despite 42.5% of adolescents were from households with home gardening, a considerably high proportion, (91.2%), of them did not consume dark green leafy vegetables and other vitamin-A rich food groups (98%). Near to three-fourth, (74.9%), of the households were food secured. Finally, nearly half 734 (47.4) adolescents were stunted, while 16.1% were thin.

Only one-tenth, 150 (9.7%), of the adolescents began menstruation between the age range of 15 to 17 years. Two-third, 1020 (65.8%), of participants took the de-worming tablet in the previous 6 months (Table 2).

**Table 2:** Dietary diversity and other nutrition and health related characteristics of adolescent girls in Dabat HDSS site, northwest Ethiopia, 2016

Variables	Response	Frequency	Percentage
Dietary diversity	Adequate DD	225	14.5
	Inadequate DD	1325	85.5
Starchy staples	No	2	0.1
	Yes	1549	99.9
Dark green leafy vegetables	No	1414	91.2
	Yes	136	8.8
Other vitamin A rich fruits and vegetables	No	1519	98.0
	Yes	31	2
Other fruits and vegetables	No	966	62.3
	Yes	584	37.7
Organ meat	No	1536	99.1
	Yes	14	0.9
Meat and fish	No	1247	80.5
	Yes	303	19.5
Eggs	No	1482	95.6
	Yes	68	4.4
Legumes, nuts, and seeds	No	289	18.6
	Yes	1261	81.4
Milk and milk products	No	1388	89.5
	Yes	162	10.5
Meal frequency	< 3 meals	69	4.5
	≥ 3 meals	1489	95.5
Purpose of home gardening	No home gardening	892	57.5
	Fully for selling	12	0.8
	Partially for selling	131	8.5
	Totally for household consumption	515	33.2
Previous alcohol intake	No	188	12.1
	Yes	1362	87.9
Current alcohol intake	No	371	23.9
	Yes	1179	76.1
Frequency of alcohol intake	Daily	55	3.5
	One time per week	434	28.0
	One times per month	543	35.0
	Two times per month	518	33.5
Stunting	Not stunted	816	52.6
	Stunted	734	47.4
Thinness	Not thin	1300	83.9
	Thinness	250	16.1
Beginning of menstruation	No	1400	90.3
	Yes	150	9.7
Menstruation started age	11–14	67	4.3
	15–17	83	5.4
Taking de-worming tablet	No	530	34.2
	Yes	1020	65.8
Taking iron folic acid tablet	No	1388	89.5
	Yes	162	10.5
Food security status	In secured	389	25.1
	Secured	1161	74.9

#### Factors associated with dietary diversity score

In the bivariate logistic regression analysis illustrated that adolescent age, occupation, educational status, and household food security had p-value of less than 0.2; accordingly passed the variable screening criteria then fitted to multivariate analysis. In the final model, only household food security status showed significant association with adolescent’s dietary diversity. Based on this finding, the odds of having adequate dietary diversity were 1.47 times (AOR = 1.47; 95% CI 1.033, 2.092) higher among adolescents from food secured households compared to adolescents living in food insecure households (Table 3).

**Table 3:** Factors associated with adequate dietary diversity among adolescent girls in Dabat HDSS site, northwest Ethiopia, 2016

Variables	Response	Dietary diversity		COR 95% CI	AOR 95% CI
		Adequate	Inadequate		
Food in security	In secured	44	344	1	1
	Secured	181	981	1.442 (1.015, 2.050)	1.470 (1.033, 2.092)*
Adolescent occupation	Farmer	10	47	1	1
	Student	204	1179	0.813 (0.404, 1.635)	0.774 (0.381, 1.574)
	Own business	11	99	0.522 (0.207, 1.316)	0.604 (0.236, 1.541)
Age of adolescents	Early (10–14)	157	829	1	1
	Middle (15–17)	62	440	0.744 (0.543, 1.020)	1.410 (0.569, 3.498)
	Late (17–18)	6	56	0.566 (0.240, 1.336)	1.116 (0.448, 2.779)
Adolescent educational status/level	Unable to read and write	10	60	1	1
	Read and write	5	33	0.909 (0.287, 2.884)	0.899 (0.282, 2.867)
	Elementary school	205	1161	1.059 (0.534, 2.103)	1.057 (0.531, 2.108)
	Secondary and above	5	71	0.423 (0.137, 1.304)	0.522 (0.164, 1.662)

* p value < 0.05

### Discussion

It well documented that woman dietary diversity is proxy indicators of micronutrient intake. The overall prevalence of adequate dietary diversity among adolescent girls was 14.5 (95% CI 12.9, 16.2). This finding is similar to the studies conducted in, Abia State, Nigeria 15.4% [13]. The high prevalence of inadequate dietary diversity in this study suggested that 85.5% of adolescent girls had poor micronutrient intake which ultimately increases their risk developing micronutrient deficiency. Adequate micronutrient (nutrient intake in general) is very critical in adolescence as it is a period of menarche and achieving substantial overall growth [11, 14]. In spite of this fact, the current finding necessitated giving critical attention to adolescent girls to lift them from the high risk of impaired growth and development, including delayed menarche and other reproductive performance measures [15].

In contrast, the reported prevalence of adequate dietary diversity was lower than earlier studies local studies in Ethiopia; Amhara Regional State (45%) and Gurage Zone, Southwest Ethiopia 26.8% [16]. Moreover, considerably high proportion of diversified diet reported urban areas of Ethiopia, specifically Gondar (75.5%) [17], Adama (54%) [18], Jimma (38.7%) [19], and Goba (45.3%) city administrations [20]. These variations in diversified diet consumption could be attributed to the characteristics of the study participants and study setting. Obviously, our study covered a wider population majority of which were rural residents (86.5%) unlike the latter reports most of which were carried out in the urban areas. A number of local studies showed better food security status of urban households than the rural households. Food security status positively affects dietary diversification or dietary intake of families. Furthermore, surpassed literacy rate and health care utilization in urban residents could also explain the improved dietary diversification among adolescent girls living in cities [21].

Our studies also lower prevalence than the studies conducted in Agarfa High School, Bale Zone, Ethiopia 80.7% [22], Goba Town, Southeast Ethiopia 45.3% [20], Gurage Zone, Southwest Ethiopia 26.8% [16], Gondar, Ethiopia 75.5% [17], Amhara Region, Ethiopia 45% [10], Adama City, Central Ethiopia 54% [18], Jimma Town, Southwest Ethiopia 38.7% [19], Southwestern Nigeria 22.5% [23]. Their difference might be due to, the majority of the participants were seen in the middle and high wealth tertile and the households who have a high economic level were a high probability to increase the diversifications of the diet. It seems that the DDS improved when the consumption of healthy food groups increased [24].

On the other hand, level of dietary diversity in our study is better than what was reported in Slum areas of Dhaka City (8.7%) in Bangladesh [10]. Slum areas are well known with impaired socio-economic status and sub-standard living conditions. These typical characteristics of slum areas adversely affect dietary habit of adolescent girls and the community at large.

In agreement to this scientific explanation, our study revealed increased odds of having adequate dietary diversity among girls living in food secured households compared to adolescents of food insecure households. The positive association between household food security and dietary diversification was also noted by different local and African reports [16, 19, 25]. It is evident that improved wealth status tends people to purchase and consume a variety of diet hence diversification of diet makes the diet more palatable and pleasant [26]. The inseparable relationship between provided good insights into the accessibility of nutrient-dense foods among the poor segment of the population. Thus, food insecurity predisposes people to relay on an undiversified diet. In general, monotonous diet has poor diet quality hence it is unlikely to get all essential nutrients in serving to consist of single food item/group [27]. Deprivations of calorie and nutrient intake for a prolonged period can erode both physical and mental capacity of growing adolescents which leads to economically less productive population [28].

This study investigated the dietary habit of adolescent girls majorly from a rural community, and a large number of both in school and out school adolescents were included which ultimately enhances the generalizability and statistical power of the study. Nevertheless, the study is not free from recall, social desirability, and misclassification biases while measuring/reporting consumption of food items and using the standardized cut-off in dichotomizing dietary diversity of participants into adequate and inadequate dietary diversity. Despite dietary diversity measurement is well documented proxy indicator of diet quality particularly for developing countries where quantification of food intake is not feasible in community-based researches, its qualitative nature could be considered as the limitation of the study.

### Conclusion

Dietary diversity among adolescent girls was considerably low in northwest Ethiopia. Food insecurity was significantly associated with undiversified diet. Given that, working to enhance household’s food security status is recommended to boost dietary diversification of adolescent’s girls.

### Limitation

The dietary diversity score was measured with 24 h recall methods and such measurement lack determination of usual habit of the foods.

## Abbreviations

HDSS: Health and Demographic Surveillance System
WHO: World Health Organization
BMI: body mass index
FANTA: Food and Nutrition Technical Assistance
AOR: adjusted odd ratio
CI: confidence interval

## References

[1] FANTA. Developing and validating simple indicators of dietary quality and energy intake of infants and young children in developing countries: summary of findings from analysis of 10 data sets. Working Group on Infant and Young Child Feeding Indicators. Food and Nutrition Technical Assistance (FANTA) Project, Academy for Educational Development (AED), Washington, D.C. 2006.

[2] WHO. (World Health Organization). Nutrition in adolescence: issues and challenges for the health sector: issues in adolescent health and development. 2005. http://whqlibdoc.who.int/publications/2005/9241593660_eng.pdf. Accessed Jan 2017.

[3] UNICEF. (United Nation Children Fund), progress for children: a report card on adolescents. UNICEF publications, New York, USA, 2012. https://www.unicef.org/publications/index_62280.html. Accessed Nov 2016.

[4] Bhutta ZA, Lassi ZS, Bergeron G, Koletzko B, Salam R, Diaz A (2017). Delivering an action agenda for nutrition interventions addressing adolescent girls and young women: priorities for implementation and research. Ann N Y Acad Sci.

[5] Rees JM, Christine MT, Adams G (1989). Nutritional influences on physical growth and behavior in adolescence. Biology of adolescent behaviour and development.

[6] Key JD, Key LL (1994). Calcium needs of adolescents. Curr Opin Pediatr.

[7] Lifshitz F, Tarim O, Smith MM (1993). Nutrition in adolescence. Endocr Metab Clin.

[8] Kennedy GL. Evaluation of dietary diversity scores for assessment of micronutrient intake and food security in developing countries. Ph.D. Thesis, University of Wagenningen, Wageningen, The Netherlands. 2009.

[9] Global Alliance, Nutrition in Adolescence, Bangladesh. 2018.

[10] Wassie MM, Gete AA, Yesuf ME, Alene GD, Belay A, Moges T (2015). Predictors of nutritional status of Ethiopian adolescent girls: a community based cross sectional study. BMC Nutr.

[11] Birru SM, Belew AK, Tariku A (2018). One in three adolescent schoolgirls in urban northwest Ethiopia is stunted. Ital J Pediatr.

[12] Gina Kennedy TB, MarieClaude Dop. Guidelines for measuring household and individual dietary diversity. Rome: FAO; 2010.

[13] Patricia OU, Ekebisi C (2016). Assessing dietary diversity score and nutritional status of rural adult women in Abia State, Nigeria. Food Sci Nutr Technol..

[14] Birru SM, Tariku A, Belew AK (2018). Improved dietary diversity of school adolescent girls in the context of urban Northwest Ethiopia: 2017. Ital J Pediatr.

[15] Mohammed AY, Tefera TB. Nutritional status and associated risk factors among adolescent girls in Agarfa high school, Bale Zone, Oromia Region, South East Ethiopia.

[16] Worku M, Hailemicae G, Wondmu A (2017). Dietary diversity score and associated factors among high school adolescent girls in Gurage Zone, southwest Ethiopia. World J Nutr Health.

[17] Birru SM, Belew AK, Tariku A (2018). One in three adolescent schoolgirls in urban northwest Ethiopia is stunted. Ital J Pediatr.

[18] Roba K, Abodo M, Wakayo T (2016). Nutritional status and its associated factors among school adolescent girls in Adama City, Central Ethiopia. J Nutr Food Sci.

[19] Melaku Y, Dirar A, Feyissa GT, Tamiru D (2018). Optimal dietary practices and nutritional knowledge of school adolescent girls in Jimma Town, South West Ethiopia. Int J Adolesc Youth..

[20] Tegegne M, Sileshi S, Assefa T, Kalu A. Nutritional status and associated factors of adolescent school girls, Goba Town, Southeast Ethiopia. Glob J Med Res Nutr Food Sci. 2016.

[21] Ethiopian Demographic and Health Survey 2016. Addis Ababa: Central Statistical Agency, The DHS Program ICF; 2017.

[22] Mohammed AY, Tefera TB (2015). Nutritional status and associated risk factors among adolescent girls in Agarfa high school, Bale Zone, Oromia Region, South East Ethiopia. Int J Nutr Food Sci.

[23] Olumakaiye MF (2013). Adolescent girls with low dietary diversity score are predisposed to iron deficiency in southwestern Nigeria. ICAN Infant Child Adolesc Nutr..

[24] Vakili M (2013). Dietary diversity and its related factors among adolescents: a survey in Ahvaz-Iran. Glob J Health Sci.

[25] Chakona G, Shackleton C (2017). Minimum dietary diversity scores for women indicate micronutrient adequacy and food insecurity status in South African Towns. J Nutr..

[26] Hoddinott J, Yohannes Y (2002). Dietary diversity as a food security indicator.

[27] Jasmine A. Parent, food security and dietary diversity amongst smallholder farmers in Haiti. 2014.

[28] FAO (2002). The right to food.

